# The complete chloroplast genome sequence of *Metabriggsia ovalifolia* W. T. Wang (Gesneriaceae), a national key protected plant endemic to Karst areas in China

**DOI:** 10.1080/23802359.2021.1884021

**Published:** 2021-03-11

**Authors:** Xiaoling Tian, Hafiz Muhammad Wariss

**Affiliations:** a The College of Humanities and Sciences of Guizhou Minzu University, Guiyang, China; b Lushan Botanical Garden, Chinese Academy of Sciences, Jiujiang, China; c Key Laboratory for Plant Diversity and Biogeography of East Asia, Kunming Institute of Botany, Chinese Academy of Sciences, Kunming, China

**Keywords:** *Metabriggsia ovalifolia*, endemic plant, chloroplast genome, phylogenomic analysis

## Abstract

*Metabriggsia ovalifolia* W. T. Wang is one of the first-class national protected plants endemic to Karst areas in China. In this study, the complete chloroplast genome was sequenced using Illumina pair-end sequencing data. The chloroplast genome was 153,333 bp in length, consisting of a pair of inverted repeats (IRa and IRb: 25,448 bp), separated by a large single copy region (LSC: 84,381 bp) and a small single copy region (SSC: 18,056 bp). The chloroplast genome encodes 131 genes, including 87 protein-coding genes, 36 transfer RNA, and eight ribosomal RNA genes. The overall GC content of the chloroplast genome was 37.55%. *M. ovalifolia* phylogenetic analysis indicated close relationship with *Oreocharis mileensis* of Gesneriaceae family. These genomic resources will be helpful for conservation of this endemic plant species.

The family Gesneriaceae, with 150 –160 genera and 3700 species, comprises perennial herbs, shrubs, or small trees, mainly distributed in the tropics and temperate regions of the world (Weber 2004; Ma et al. 2011). In China, it has 58 genera and 463 species, with 27 endemic genera and 375 endemic species (Li and Wang 2004). In addition to being ornamental, this family harbors several medicinal species which are used in traditional Chinese medicines (Ma et al. 2011). *Metabriggsia* W. T. Wang is an endemic genus of China belongs to subfamily Didymocarpoideae and Trichosporeae tribe of Gesneriaceae, consists of only two species. *Metabriggsia ovalifolia* W. T. Wang is a perennial herb, endemic to Guangxi and Yunnan provinces of China (Yao et al. 2016). Due to its small population size and restricted distribution range, *M. ovalifolia* was listed as, ‘first grade rare and endangered species in China’ and also included among the plant species with extremely small population (PSESP), an emergency rescue plan for threatened species in China (Ren et al. 2012; Yao et al. 2016). Hence, it is vital to protect the germplasm resources of *M. ovalifolia*. In this study, we sequenced the chloroplast genome of *M. ovalifolia* to provide valuable genomic resources to expedite conservation of this endemic species.

Plant material was collected from Guangxi Institute of Botany, Guangxi Zhuang Autonomous Region (N25°07′63.1″, E110°30′35.3″, 190 m a. s. l.), Chinese Academy of Sciences, Guilin, Guangxi, China and voucher specimen (MYP2020010) was deposited in the Herbarium of Guangxi Institute of Botany (IBK). These plants were introduced from Libo County, South of Guizhou province, China. Genomic DNA was isolated using Tiangen plant genomic DNA kits (Tiangen Biotech, Beijing, China) and sequenced on the Illumina Hi-Seq 2500 platform. A total of 6 Gb of 150 bp pair end raw reads were obtained and used for assembling the chloroplast genome using GetOrganelle pipeline (Jin et al. 2019). The chloroplast genome annotation was performed using *Primulina huaijiensis* (NC_036413) as a reference genome following Geseq and further manually verified and visualized in Geneious Prime v.2019.1.3 (Kearse et al. 2012; Tillich et al. 2017). Finally, annotation was corrected with CPGAVAS2 (Shi et al. 2019) and the chloroplast genome with accession number MW145180 was deposited in the NCBI GenBank. 

*M. ovalifolia* chloroplast genome was 153,333 bp in length, including a large single copy (LSC) region of 84,381 bp and a small single copy (SSC) region of 18,056 bp, with a pair of inverted repeat (IR) regions of 25,448 bp each. The overall GC content of the chloroplast genome was 37.55%. In total, the chloroplast genome contained 131 genes, consists of 87 protein-coding genes, 36 transfer RNA genes, and eight ribosomal RNA genes.

The complete chloroplast genome sequence of *M. ovalifolia* and previously published 11 chloroplast genomes from the Gesneriaceae family were used to investigate the phylogenetic relationships using *Streptocarpus ionanthus* as outgroup. A maximum-likelihood tree with 1000 bootstrap replicates was inferred by RAxML-HPC2 Workflow on XSEDE (v.8.2.12) using the CIPRES online portal (Miller et al. 2010) based on alignments created by MAFFT with default settings (Katoh and Standley 2013). The reconstructed phylogeny (Figure 1) revealed that *M. ovalifolia* grouped with *Oreocharis mileensis*. Our results provide fundamental genetic resources for conservation and future evolutionary studies of this endemic species of China.

**Figure 1.**
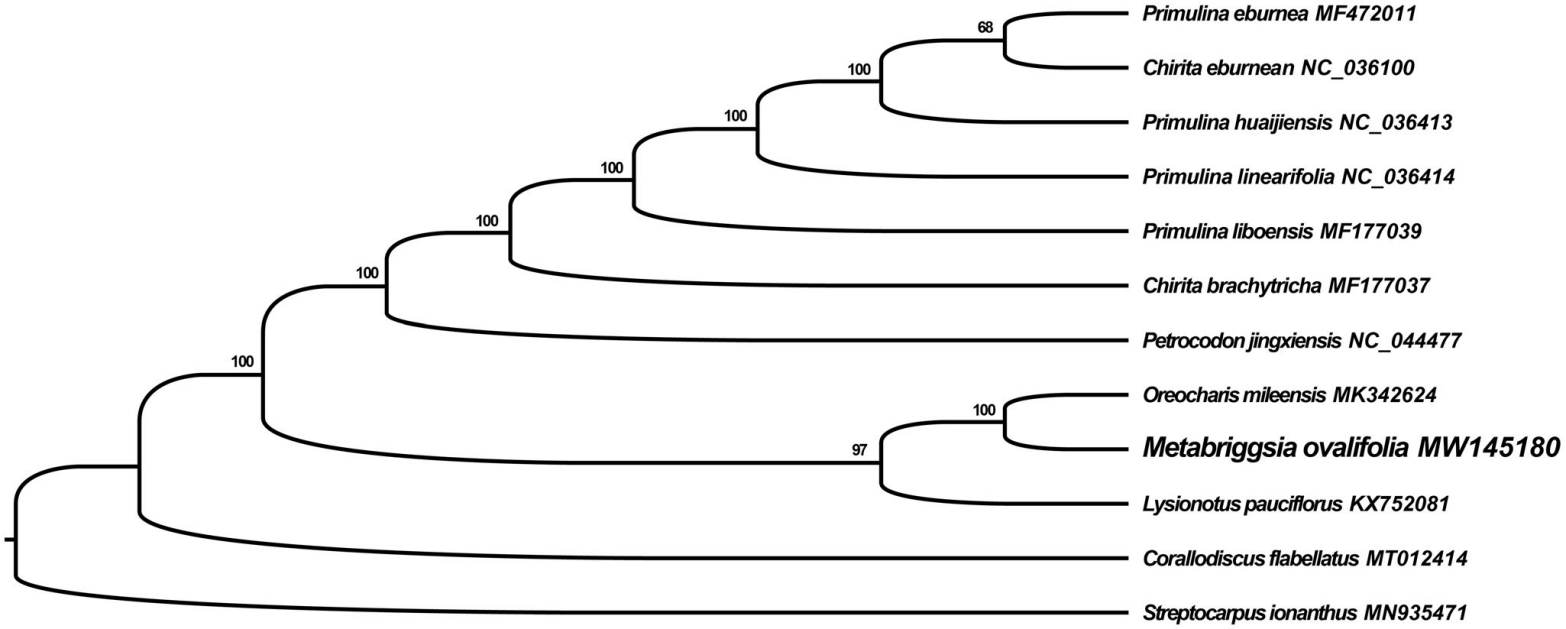
Maximum-likelihood phylogenetic tree based on 12 complete chloroplast genome sequences. The NCBI accession number for each species is given after its scientific name and the number at each node indicates bootstrap support value.

## Data Availability

The genome sequence data that support the findings of this study are openly available in GenBank of NCBI at https://www.ncbi.nlm.nih.gov/ under the accession no. MW145180. The associated BioProject, SRA, and Bio-Sample numbers are PRJNA686484, SRR13277476, and SAMN17118398, respectively.
